# Concept analysis of human dignity in patient care: Rodgers' evolutionary approach

**Published:** 2018-04-18

**Authors:** Maliheh Kadivar, Marjan Mardani-Hamooleh, Marjan Kouhnavard

**Affiliations:** 1 *Professor, Division of Neonatology, Department of Pediatrics, Children’s Medical Center, Tehran University of Medical Sciences, Tehran, Iran. *; 2 *Assistant Professor, Department of Psychiatric Nursing, Iran University of Medical Sciences, Tehran, Iran. *; 3 *Pediatrician, Children’s Medical Center, Tehran University of Medical Sciences, Tehran, Iran.*

**Keywords:** *Human dignity*, *Patient care*, *Ethics*, *Rodgers’ evolutionary approach*

## Abstract

Human dignity (HD) in patient care is an important concept in clinical ethics that has various definitions in existing literature. This study aimed at analyzing the concept of HD in patient care. To this end, Rodgers' evolutionary concept analysis was used. For this purpose, scientific databases PubMed, Elsevier, ScienceDirect, Scopus, OVID, Web of Science, CINHAL, IRANDOC, Google Scholar, Magiran, SID and IranMedex were searched fusing the words “human dignity”, “patient care” and “ethics”. The main criterion for inclusion in the final analysis was the literature published in English and Persian from 2006 to 2016 in online scientific journals within the context of health care disciplines. Ultimately, 21 articles were selected for the study. The attributes of the concept under study were identified in two areas of individual HD and social HD. Antecedents included facilitators and threats, and the consequences consisted of both favorable and unfavorable consequences. HD forms the essence of patient care and is a value-based and humanistic concept based on respect for the integrity of human beings and their beliefs. This concept, with its holistic approach to humans, takes into account all stages of disease, old age and the end of life period. HD in patient care is influenced by cultural, social, spiritual and religious factors, and with its justice-based approach emphasizes equality of all patients and extends patient care to all areas of society rather than restricting it to hospital settings. In this study, a clear definition of HD is introduced.

## Introduction

Human dignity (HD) has been emphasized throughout history by philosophical schools and various religions, especially Islam. The concept is rooted in ancient schools and religions, Middle Ages, and modern times. HD follows all the principles of biomedical ethics (1). On the one hand, maintaining HD is one of the primary rights of human beings (2). According to Holy Quran, God breathed His spirit into man, and therefore man has a unique nature. God created the entire world for man so that he could enjoy the numerous bounties, and He dignified him as He ordered the angels to bow before him. These matters clarify the intrinsic dignity of humankind (3). In fact, HD is an innate value that has been vested in people because they are human. (2) According to the Iranian constitution, HD is one of the basic rules of the Islamic Republic, and the government should therefore offer health services to all nationals based on respect and observance of HD (4).

Privacy is one of the most important dimensions of HD, and healing environments are among the places where privacy may be threatened or invaded. Maintaining HD creates pleasant feelings in patients that should not be disrupted through care provision (5). In fact, it is essential to preserve patient privacy in order to establish effective communication between patients and care staff, and preserve the former’s peace of mind. If this is neglected, consequences may include anxiety, aggression, partial withholding of the history of the disease, refusal to undergo physical examinations, and a general decrease in the quality of care services (6). Some factors that may threaten the dignity of hospitalized patients are: an inability to perform important roles in life, the feeling that life is meaningless, lack of support from friends and the health care staff, a sense of uncertainty, and concern for the future (7). Furthermore, whenever patients’ autonomy is compromised, respect for their dignity decreases (8).

It can be stated that maintaining HD is one of the ethical goals in care (9). In other words, preserving HD is among the most significant ethical requirements in medical sciences, especially nursing, so that respect for patients’ HD is widely emphasized in nursing standards and lies at the heart of care (10). According to these ethical considerations, since promoting the quality of clinical services is now at the top of the medical care pyramid, maintaining patient privacy has become more important (1). Nevertheless, while the concept of HD in care is closely interlinked with medicine and medical sciences, the existing knowledge about this concept is not well-defined and there is no comprehensive definition for it. In the field of research, the paucity of studies on this concept has limited its utilization to some extent. Therefore, considering that a single definition of the concept of HD in patient care is not available and its application is still unclear, the present study aimed to analyze the concept of HD in patient care using Rodgers’ evolutionary approach (REA).

## Method

This study was a qualitative concept analysis of HD in patient care using REA (11), which included the definition of the concept as well as its alternative statements and terms. The study involved the determination and selection of scope for data collection, the collection of data on the characteristics of the concept according to context variability’s such as social and cultural changes, interdisciplinary and temporal changes, and the expression of an appropriate case in terms of the concept and determination of the hypotheses and analytical implications for further development of the concept (11).

**Fig.1:**
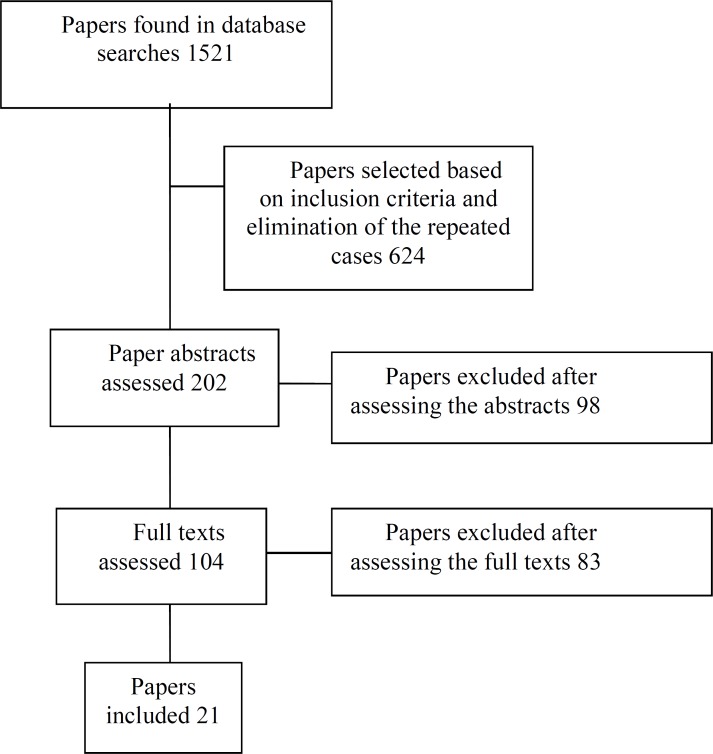
Summary of the search

At first, scientific databases PubMed, Elsevier, Science Direct, Scopus, OVID, Web of Science, CINHAL, IRANDOC, Google Scholar, Magiran, SID and Iran Medex were searched using the headings defined in MeSH system that included “human dignity”, “patient care” and “ethics” in the title, abstract and full text of the published articles. A summary of the search is shown in Figure 1.

The inclusion criterion in the final analysis was the literature published in English and Persian in online scientific journals within the context of health care disciplines between 2006 and 2016. Literature analysis was conducted using content analysis. The information units included either related words and sentences, or answers given to the following questions: How is HD defined in patient care? How is HD formed in patient care? What factors play a role in maintaining HD in patient care? What are the consequences of not respecting HD in patient care?

The papers were classified according to the conceptual analysis characteristics that were considered in this study. In order to ensure objectivity, validity and reduction of bias, the analysis process was carried out by two PhD nurses who were not members of the research team, but had clinical records and were familiar with REA.

## Results

After reading the articles, the appropriate points and the attributes, antecedents, consequences, related concepts, surrogate terms, and concept definition were extracted. Next, in each section, the data were reviewed several times so that the researcher could become immersed in them and extract key points and labels to provide clear descriptions of each aspect of the concept. Finally, the inductive analysis of information on the concept was carried out and the themes were identified (Table 1).

.

**Table1:** Characteristics of the articles

Reference no.	Researcher/Year	Attributes	Antecedents	Consequences
5	Coventry/2006	Dignity is an inner feeling of being good, personal valuation and self-esteem. Preserving HD is not limited to clinical environments and involves all patients in various areas of the community. HD is influenced by the relationship with others.	In order to provide care services while preserving HD, one needs to get help first and foremost. In order to preserve HD, autonomy of patients and respect for their individuality must be taken into account. Providing care services while preserving HD requires truth- clarification, observance of patients’ rights, and equity in care provision.	Taking HD into account leads to providing care that considers patients’ values and beliefs and their cultural diversity. Providing health care services by preserving HD brings about patients’ respect and strengthens their independence.
14	Pleschberger/2007	Dignity is rooted in people’s beliefs. Dignity is influenced by social relationships, including those with family and friends.	Disease and the need for care threaten HD.	
24	Slettebø et al./2009		Providing patients with relevant information about the disease helps them maintain their HD. If patients are neglected by staff or are faced with staff who lack adequate knowledge, their HD will be threatened.	The sense of HD improves patients’ ability to cope with their disease and gives them a sense of meaningfulness in life.
9	Lin et al./ 2011	HD is based on the values, attitudes and perceptions of individuals. HD is a cultural concept.	Preserving information about the disease and meeting the needs of patients will enhance their dignity.	Preserving HD increases patients’ satisfaction with care services.
2	Ebrahimi et al./2012		Taking patients’ privacy into consideration and effective communication with care workers lead to the preservation of HD. The paucity of supplies such as blankets and linen in the ward, which prevents patients from using the existing facilities and leads to admission into a dirty and noisy ward, adversely affects their HD.	
27	Lindwall et al./ 2012		HD is promoted in patients through nurses’ moral responsibility in taking care of patients. Restricting patients undermines their HD.	
26	Bagheri et al./ 2012		Having authority in interpersonal relationships plays a role in promoting HD.	
28	Holmberg et al./2012			Preserving HD improves patients’ self-esteem and their trust in nurses.
15	Hall et al./2013	HD forms the essence of patient care.		Preserving HD helps patients with their lives in the future. Violation of HD in patient care leads to spiritual and psychological distress and loss of the will to survive.
19	Hamooleh et al./2013	HD is an important concept in care that considers the integrity of patients and sees them as human beings to the very last moment of their life.	HD-based care services provision comprises respect for patients, paying attention to their values and empathy. HD is achieved by respecting patients’ values.	
17	Lohne et al./2014	HD is a complex concept combined with elements such as respect, assurance and security for patients.		
12	Hall et al./ 2014	HD is an important aspect of a person's quality of life structure.	Preserving HD in old age is possible through respect, observing their privacy and autonomy, and considering elderly people as human beings.	
22	Cheraghi et al./2014	HD is based on social, cultural, religious and spiritual factors.	Respecting patients and providing patient-centered care are effective in providing HD-based care	
10	Manookian et al./2014	All humans have dignity and must always be respected, but patients deserve special attention in this respect.	From patients’ point of view, factors promoting patients’ HD include respect for human nature and the rights of patient’s companions.	
18	Cheraghi et al./2015	Taking HD into consideration means regarding patients as human beings and not as objects. In patient care, HD is characterized by equality of all human beings.		Taking HD into consideration leads to providing care services based on kindness and affection.
16	Papastavrou et al./2016	Respect for HD is one of the essential parts of care.	Considering patients as unique human beings, paying attention to their preferences, confidentiality, and preserving their privacy and autonomy are factors that help promote HD.	
20	Gysels et al./2016	HD is a fixed value in end-of- life care.	Having a disease is a threat to people’s dignity.	
25	Borhani et al./2016		Paying attention to the needs of patients at the time of admission and after discharge, and the proper nurse-patient relationship are effective factors on preserving patients’ dignity.	Preserving HD will make patients feel that they matter to the care system.
13	Bagheri et al./ 2016	Dignity is an intrinsic characteristic of human existence that is experienced mentally.	Violation of patient privacy, lack of patients’ participation in the decision-making processes and lack of support threaten patients’ dignity.	
21	Granero-Molina et al./2016	HD is one of the pillars of good death for patients at the end of life stage.	Lack of proper space in the ward to provide patients with end of life care and lack of palliative care services lead to a decline in HD.	
23	Høy et al./ 2016	Preserving HD is an important goal in caring for the elderly people.		Preserving HD in providing care services causes patients to have fewer problems adapting to the community. Violation of HD harms patients.

**A. Attributes**

Attributes are the elements that identify the concept under study (11). The two following attributes were identified in connection with the concept of HD in patient care:

*A. 1. Individual HD*

The concept of individual HD is characterized by those aspects of patient care that acknowledge patients’ individuality and integrity, and respect their dignity as human beings throughout the life stages. HD is an important component of the individual quality of life structure (12) and is an intrinsic characteristic of human existence that is perceived mentally (13). The concept of dignity is rooted in people’s beliefs (14) and is based on their values ​​and perceptions (9). Dignity is a kind of inner feeling of being good, personal valuation, and self-esteem (5). All humans have dignity and must be respected in any case, but patients deserve special attention in this regard (10). Since HD forms the basis of patient care (15), respect for HD has been taken into account as one of the essential components of patient care (16). HD is a complex concept combined with elements such as respect, assurance and security for the patient (17). Attention to HD means considering the patient as a human being and not an object (18). Thus, HD is a meaningful concept in care that makes it possible to respect patients’ integrity and regard them as human beings till the end (19). Accordingly, HD is a fixed value in end-of-life care (20) and is one of the pillars of good death for patients in terminal stages (21).

*A. 2. Social Dignity*

The concept of HD in the social domain includes attributes that reflect the social and cultural factors involved in HD. This attribute also emphasizes its holistic aspect and the fact that it can be influenced by communication. Dignity can be affected by social relationships, including those with family and friends (14). HD is a cultural concept with a religious and spiritual basis (22), and in patient care it is characterized by considering all human beings equal (18). On the other hand, preservation of HD is not restricted to clinical settings and patients who are admitted to hospitals; it covers all patients in various areas of the community (5), and is an important goal in caring for elderly people living in sanatoriums (23).

**B. Antecedents**

Antecedents are the preconditions of the concept under study and affect the occurrence of the concept (11). In the present study, they have been identified as facilitators and threats.

*B. 1. Facilitators*

Facilitators include items that help to provide, preserve, and promote HD in patient care. In order to preserve HD while offering care services, first and foremost, one needs to get help (5). Effective factors in providing HD-based care include respect for the patient and provision of patient-focused care (22). HD is shaped by respecting patients’ values (19). To preserve HD, autonomy of patients and respect for their individuality must be taken into account. Care that is based on preservation of dignity relies on patient autonomy, truth-telling, respect for patients’ rights and equity in care provision (5). With regard to the privacy of patients admitted in hospitals, some factors that may ensure the preservation of HD are effective communication with caregivers (2), and receiving relevant information about the disease (24). HD-focused care services entail empathy and respect for patients’ values (19). Considering the needs of patients at the time of admission and after discharge, and proper nurse-patient relationship are effective in HD preservation (25). In old age, preservation of HD is possible through autonomy of elderly people and regarding them as human beings (12). Factors that can promote patients’ dignity include recognition of their authority in interpersonal relationships (26), respect for human nature and the rights of patients’ companions (10), keeping information about the disease confidential, and meeting the needs of patients (9). Other effective factors on dignity in patient care include considering the patient as a unique person, confidentiality and paying attention to his preferences (16). In general, HD is promoted through nurses’ moral responsibility in taking care of patients (27).

*B. 2. Threats*

Threats refer to factors that jeopardize HD in patient care and may be patient-related or organizational. As a general rule, having a disease can threaten people’s dignity (14, 20). Violation of privacy, lack of patient participation in the decision-making process, and insufficient or non-existent support can compromise patients’ dignity (13). In addition, being neglected by the medical team or encountering staff who lack adequate knowledge pose a threat to patients’ HD (24). Moreover, restricting patients, which includes emergency measures in sectors such as the psychiatric ward, undermines their HD (27). Other factors that lead to a reduction in patient dignity include lack of suitable hospital space in end-of-life care, and a shortage of palliative care services (23). Paucity of supplies such as blankets and linen in the wards prevents patients from using the existing facilities, and admission to dirty and noisy wards adversely affects their HD (2). 

**C. Consequences**

Consequences refer to events that follow the emergence of a concept (11). In this study, the consequences of the concept of HD in patient care were divided into two categories: favorable and unfavorable.

*C. 1. Favorable Consequences*

Favorable consequences have positive effects on HD in patient care. Taking HD into consideration leads to respect for patients’ values, beliefs and cultural diversity in care provision. Preservation of dignity enhances patients’ sense of respect and autonomy (5) and helps them feel worthy of the care system (25); moreover, it leads to care services that are based on kindness and affection (16), increases patients’ satisfaction with the care process (9) and promotes patients’ self-esteem and trust in nurses (28). Preservation of patients’ dignity helps them adapt to the community with greater ease (23), improves their ability to cope with the disease and gives them a sense of meaningfulness in life (24). Preserving HD helps patients with their future life, especially in the case of cancer patients (15).

*C. 2 Unfavorable Consequences*

Just as preservation of HD in patient care has favorable consequences, paying no attention to HD has unfavorable outcomes. Violation of HD harms the patients (23), and in the case of cancer patients for instance, may lead to spiritual and psychological distress and loss of the will to survive (15).

**Related Concepts and Surrogate Terms**

Related concepts include some of the relationships included in the main concept, but do not comprise all of its characteristics (11). The term “patient dignity” in this study was most closely related to the concept under study in the analysis of the relevant literature. Surrogate terms refer to the expression of the concept through words and statements other than the concept selected for the study (11). It was established in the analysis process that the concept of HD in patient care could be replaced by terms such as *dignity in care* and *dignified care*.

**Environmental Context**

Environmental contexts are the fields and situations in which an issue is utilized (11). Healthcare and clinical settings are the most general environments utilizing the concept of HD in patient care.

**Model Case**

Model cases help to identify the important characteristics of the concept under study in the main context and lead to clarity and a more effective use of that concept (11). 

M. is a 34-year-old woman who has referred to the oncology department with a gastric cancer diagnosis and needs to have a part of her cancerous stomach removed. She is accompanied by her brother. The patient cries and is afraid of the future. She claims that in her opinion, life is worthless and this is the end of the world for her. The nurse realizes that the patient is suffering from emotional distress due to cancer and needs help. So first, the nurse lets her cry and then she asks the ward staff to admit her with respect. Next, the nurse goes to the patient’s room and, gives her the facts about cancer and its treatments, and proceeds to provide empathic care in order to reduce the emotional distress of the patient. She also tries to help the patient to go on with her life by reinforcing the meaning of life for her. The nurse believes that providing palliative care for all cancer patients as well as giving special attention to them are essential for improving their quality of life to the last moment. Over the entire care period, the nurse feels responsible for keeping the patient’s information confidential and respecting her autonomy.

**Contrary Case**

Ms. B., a 57-year-old woman with heart failure, was admitted to the cardiology unit of a hospital at night. She had no companions. That night the unit was quite busy, and the patient used the nurse call button frequently. Due to her heavy workload that night, the nurse did not pay attention to the calls. Another patient’s visitor informed the nurse that B. was unable to go to the bathroom by herself. This annoyed one of the nurses, who went to B.’s room and told her rudely that she should have brought in a companion because the unit was busy and service was limited. At the same time, the nurse ordered a serviceman to give her a bedpan. The patient refused to use the bedpan, but the nurse did not allow her to leave her bed as a precautionary measure. The patient started to cry because of the nurse’s negligence and disrespect, and the threat to her autonomy. The nurse, however, left to go back to her station while the patient was still crying. 

In this case the study concept cannot be covered, and is in fact recognized by paradoxical characteristics. 

**Related Case**

Related cases do not reflect all the aspects of the concept, but are related to it, and in comparison with the contrary case are closer to the study concept and could even be listed in the same network.

Mr. F. was a 29-year-old man diagnosed with Schizophrenia and was scheduled to receive electro convulsive therapy. Because of the psychotic nature of the disease, he could not sign his consent. His father decided that his son should undergo the procedure based on his pre-disease beliefs about health and out of respect for his ideas.

**Borderline Case**

Cases that do not improve knowledge or contribute to science in a specific field are considered borderline cases. 

Ms. T, is an 87-year-old woman who can take care of her personal needs if she does not have a backache or sore feet. She has a good relationship with her young son, and asks for his help when she cannot do her self-care. One time she could not get out of bed and her son was not there. She asked another patient for help and fell on the ground. Her nurse was informed and said, “What you did was wrong, and you should have informed us”. The patient insisted that she had done the right thing decisively and with self-respect, and after her son arrived, she also told him that she believed she was right.

**Identifying Assumptions of the Concept Analysis**

This stage provides a debate opportunity to utilize the findings of an analysis (11). The concept analysis showed both individual and social HD to be important issues in the definition of the concept. The favorable and unfavorable consequences associated with HD in patient care are affected by a series of simplifiers and threats. In addition, HD is a connecting issue between the advances and consequences, and without the main concept, the connection is disrupted; as a result, it becomes impossible to link the advances and consequences with each other.

## Discussion

An analysis of the concept of HD in patient care has several dimensions. Our findings showed that this concept includes a range of attributes, antecedents, and consequences.

Our study revealed that the concept under study has individual and social attributes. Individual attributes indicate that it is necessary to raise public awareness about this issue, especially among patients, because HD is the essence of patient care (15). The results of the studies conducted in the United States (5), Iran (13), Taiwan (9), Germany (14) and Cyprus (16) demonstrate that human beings have an inherent dignity, and HD in patient care is a complex concept that includes elements such as respect for human individuality, values and beliefs. In addition, a review of literature on research conducted in England (12), Spain (21) and Iran (19) shows that HD in patient care includes an important aspect of quality of life structure and should be preserved as a value throughout life.

Evaluation of the social attributes of the concept under study indicates a sort of justice-centered approach. The results of an Iranian study revealed that preservation of HD is intertwined with the notion of equality of all patients (18). HD encompasses all patients in various areas of the society in addition to hospitals (5). Therefore, it is an important goal in caring for seniors living in elderly sanatoriums across Scandinavian regions (23). In its social dimension, HD is influenced by relationships with others. The results of a study in Germany showed that the concept of dignity could be influenced by social relationships, including those with family and friends (14). The results of a study in Taiwan demonstrated that another social feature of the concept is its cultural origins (9). From the perspective of Iranian patients, HD is based on social, cultural, religious and spiritual factors (22). This indicates the need to consider the culture of patients when providing dignified care, because their cultural beliefs and perceptions can affect this type of care. Indeed, care is a context-based concept influenced by cultural settings and the values ​​governing societies. Consequently, those receiving care in the same cultural context will have common characteristics that affect the process of providing dignified care, which is characterized by observance of HD.

Considering the findings on the features of this concept, we can define HD in patient care as follows: “*HD is the essence of patient care. HD in patient care is a value-based and humanistic concept that demands respect for the integrity of human beings and their beliefs. This concept has a holistic approach to humans and takes into account the stages of disease, old age and the end of life. HD in patient care is influenced by cultural, social, spiritual and religious factors, and views all the patients equally with a justice-based approach. Accordingly, HD focuses on patient care in all social arenas and is not specific to the hospital environment*”.

Based on the antecedents of the concept under study, there are facilitators and threats to providing dignified care to patients. The facilitating antecedents showed that in order to provide care and preserve patient dignity, one needs to receive help first. In addition, the patient’s autonomy and respect for his individuality must be considered. Dignified care is possible by observing factors such as truthfulness, patients’ rights, and equity in care services (5). A number of studies have focused on the facilitating antecedents of this concept from the perspective of patients in general or patients with a particular disease. In this regard, a review of Iranian literature showed that from the viewpoint of hospitalized patients, some factors that lead to the preservation of HD are: respect for patient privacy, effective communication with caregivers, respect for the human nature and the rights of companions, provision of patient-centered care, proper nurse-patient communication, and paying attention to the needs of patients at the time of admission and after discharge (2, 22, 25). From the viewpoint of patients admitted to Taiwan hospitals, preserving disease information and responding to patients’ needs helped promote their dignity (9). A study in Norway showed that from the perspective of patients with head injury, receiving proper information about the disease would help preserve their HD (24). In one study conducted in Iran on patients with heart failure, factors such as having authority in interpersonal relationships contributed to the promotion of HD (25).

A number of studies have dealt with the facilitators of the concept from nurses’ point of view. From the perspective of Swedish nurses, HD in patient care improves with the moral responsibility of nurses (27). In a study conducted in Cyprus, nursing students believed factors promoting dignity in patient care include perceiving the patient as a unique person, taking into account patients’ preferences, and respecting their privacy and autonomy (16). From the perspective of Iranian nurses on caring for cancer patients, HD is shaped by paying attention to patients’ values, providing care focused on preserving their dignity, respecting them, ​​and empathy (19). According to nurses who care for the elderly in England, preservation of HD in old age is possible through respect, considering the elderly as human beings, and observing their privacy and autonomy (12).

Other facilitators consider patients with chronic illnesses such as cancer and acute illnesses such as strokes. It can be stated that the preservation and promotion of HD in patient care is not limited to acute or chronic illnesses and covers all states of disease.

The other antecedents of the concept under study are factors that threaten the concept. These antecedents indicate that having a disease threatens HD (14, 20). In addition, issues such as violation of patients’ privacy, lack of participation in the decision-making process and lack of patient support lead to a threat to their dignity (13). Studies have examined conceptual threats for both patients and clinical practitioners, namely doctors and nurses. The opinions of practitioners focused both on the general environment of the hospital and on the particular ward. From the viewpoint of hospitalized patients in Iran, lack of supplies such as blankets and linen in the wards makes it impossible for patients to use the existing facilities properly, and their admission to noisy and dirty wards undermines their HD (2). Norwegian patients believed that their dignity is threatened if they are neglected by medical staff or encounter employees with inadequate knowledge (24). A study in Spain showed that from the viewpoint of doctors and nurses, a shortage of appropriate space in the emergency department to provide end-of-life care for patients and lack of palliative care services would reduce patients’ dignity (21). From the perspective of Swedish nurses, limiting the patient, as one of the emergency measures in psychiatric wards, threats patients’ HD (27).

Literature review revealed that advances in the concept of dignity in patient care presented as facilitators and threats are important from the point of view of both patients and medical professionals. Furthermore, concept analysis of the antecedents indicates that HD in patient care is not in a steady state and may be promoted or threatened under different circumstances. Therefore, caregivers should be more aware of the factors that threaten the concept, and show greater moral sensitivity. Other findings of the study showed that the consequences of the concept of HD in patient care could be either favorable or unfavorable.

Concerning the favorable consequences, attention to HD in care provision leads to respect for patients’ values ​​and beliefs and their cultural diversity, and helps preserve their dignity and improve their autonomy (5). According to hospitalized patients and nurses in Iran, preservation of HD helps patients feel that they are considered valuable in the care system (25). From the viewpoint of Iranian patients, attention to dignity in patient care leads to the provision of services based on compassion and love (18). For Taiwanese patients, preserving HD led to increased satisfaction with care services (9). From the perspective of Swedish patients, promotion of HD in homecare nursing was shown to improve patients’ self-esteem and their trust in nurses (28). Findings of a study in the Scandinavian region indicated that dignified care helps the elderly to better adapt to the community (23). Patients in Norway who had suffered stroke found that preservation of HD improved their ability to cope with the illness and gave their life a sense of meaningfulness (24). From the viewpoint of cancer patients and their family caregivers in the UK, preserving HD helped patients with their life in the future (15). These findings suggest that the desired consequences of dignified care are social, cultural, moral, mental and spiritual.

The results emphasized the importance of addressing factors that affect preservation and promotion of HD in patient care, since ignoring these issues leads to unfavorable consequences in this regard. The findings of a Scandinavian study showed that violation of dignity of the elderly increases their vulnerability (23). According to a study in England, cancer patients and their family caregivers believed that neglecting HD in care leads to spiritual and psychological distress and loss of the will to survive (15). In general, the results of the concept analysis indicated that in patient care, preservation and promotion of HD is necessary and its violation leads to unfavorable consequences. In order to prevent the unfavorable outcomes, threatening antecedents must be prevented. To this end, caregivers should be more sensitive to identifying the unfavorable consequences of HD violation in patient care and, while managing them properly, reduce their incidence and subsequent consequences.

It should be noted that one of the limitations of this study was our inability to use full text papers published in non-English languages, and this may have resulted in presenting an incomplete image of the examined concept. Additionally, analysis of the concept of HD in the care of a specific disease was not performed, and therefore other researchers are encouraged to perform a similar study on a particular illness.

## Conclusion

In this study, a clear definition of HD was introduced. Through this definition, health policy makers can revise effective care frameworks in clinical settings. Since moral patient care with emphasis on HD preservation is an essential component of care provision, it can be concluded that attention to the attributes, antecedents and consequences identified in this study can be useful in better identification of this concept. In addition, awareness of this concept can lead to the promotion of its status, importance and application for medical professionals in the field of clinical ethics as they are deeply involved with the care of patients. Through identification of the concept attributes, the medical team will be able to provide a suitable platform for further development of its facilitating antecedents and, as a result, will have more exposure to the favorable consequences of HD care. In addition, recognizing the features of the concept helps the team to reduce the unfavorable consequences resulting from violation of HD in patient care by preventing the occurrence of threatening antecedents. The findings can help develop theories in this regard through clarification of the concept under study. Finally, the findings could be a guide for practitioners in the field of medical ethics in order to incorporate this important concept into educational programs for the students. 

## References

[1] Aramesh K (2007). Human dignity in Islamic bioethics. IJAAI.

[2] Ebrahimi H, Torabizadeh C, Mohammadi E, Valizadeh S (2012). Patients' perception of dignity in Iranian healthcare settings: a qualitative content analysis. J Med Ethics.

[3] Lavasani SMH, Kalantarkousheh SM (2013). The roots of human dignity according to Quranic verses. Australian Journal of Basic and Applied Sciences.

[4] Salehi HR (2013). Human dignity from the viewpoint of Iranian law. J Bioeth Inq.

[5] Coventry ML (2006). Care with dignity: a concept analysis. J Gerontol Nurs.

[6] Hemati Z, Ashouri E, AllahBakhshian M (2016). Dying with dignity: a concept analysis. J Clin Nurs.

[7] Mehdipour-Rabori R, Abbaszadeh A, Borhani F (2016). Human dignity of patients with cardiovascular disease admitted to hospitals of Kerman, Iran, in 2015. J Med Ethics Hist Med.

[8] Gennip IEV, Pasman HRW, Oosterveld-Vlug MG, Willems DL, Onwuteaka-Philipsen BD (2016). How dementia affects personal dignity: a qualitative study on the perspective of individuals with mild to moderate dementia. The Journal of Gerontology.

[9] Lin YP, Tsai YF, Chen HF (2011). Dignity in care in the hospital setting from patients' perspectives in Taiwan: a descriptive qualitative study. J Clin Nurs.

[10] Manookian A, Cheraghi MA, Nasrabadi AN (2014). Factors influencing patients' dignity: A qualitative study. Nurs Ethics.

[11] Rodgers BL, Knafl KA (2000). Concept Development in Nursing: Foundation, Techniques and Application.

[12] Hall S, Dodd RH, Higginson IJ (2014). Maintaining dignity for residents of care homes: a qualitative study of the views of care home staff, community nurses, residents and their families. Geriatr Nurs.

[13] Bagheri H, Yaghmaei F, Ashktorab T, Zayeri F (2018). Test of dignity model in patient with heart failure. Nurs Ethics.

[14] Pleschberger S (2007). Dignity and the challenge of dying in nursing homes: the residents' view. Age Ageing.

[15] Hall S, Goddard C, Speck PW, Martin P, Higginson IJ (2013). "It makes you feel that somebody is out there caring": a qualitative study of intervention and control participants' perceptions of the benefits of taking part in an evaluation of dignity therapy for people with advanced cancer. J Pain Symptom Manage.

[16] Papastavrou E, Efstathiou G, Andreou C (2016). Nursing students' perceptions of patient dignity. Nurs Ethics.

[17] Lohne V, Rehnsfeldt A, Raholm MB, Lindwall L, Caspari S, Saeteren B (2014). Family caregivers' experiences in nursing homes: narratives on human dignity and uneasiness. Res Gerontol Nurs.

[18] Cheraghi MA, Manookian A, Nikbakht Nasrabadi A (2015). Patients' lived experiences regarding maintaining dignity. J Med Ethics Hist Med.

[19] Hamooleh MM, Borimnejad L, Seyedfatemi N, Tahmasebi M (2013). Perception of Iranian nurses regarding ethics-based palliative care in cancer patients. J Med Ethics Hist Med.

[20] Gysels M, Reilly CC, Jolley CJ (2016). Dignity through integrated symptom management: lessons from the breathlessness support service. J Pain Symptom Manage.

[21] Granero-Molina J, Díaz-Cortés Mdel M, Hernández-Padilla JM, García-Caro MP, Fernández-Sola C (2016). Loss of dignity in end-of-life care in the emergency department: a phenomenological study with health professionals. J Emerg Nurs.

[22] Cheraghi MA, Manookian A, Nasrabadi AN (2014). Human dignity in religion-embedded cross-cultural nursing. Nurs Ethics.

[23] Hoy B, Lillesto B, Slettebo A (2016). Maintaining dignity in vulnerability: a qualitative study of the residents' perspective on dignity in nursing homes. Int J Nurs Stud.

[24] Slettebo A, Caspari S, Lohne V, Aasgaard T, Naden D (2009). Dignity in the life of people with head injuries. J Adv Nurs.

[25] Borhani F, Abbaszadeh A, Rabori RM (2016). Facilitators and threats to the patient dignity in hospitalized patients with heart diseases: a qualitative study. Int J Community Based Nurs Midwifery.

[26] Bagheri H, Yaghmaei F, Ashktorab T, Zayeri F (2012). Patient dignity and its related factors in heart failure patients. Nurs Ethics.

[27] Lindwall L, Boussaid L, Kulzer S, Wigerblad A (2012). Patient dignity in psychiatric nursing practice. J Psychiatr Ment Health Nurs.

[28] Holmberg M, Valmari G, Lundgren SM (2012). Patients' experiences of homecare nursing: balancing the duality between obtaining care and to maintain dignity and self-determination. Scand J Caring Sci.

